# Recurrent circuits encode *de novo* visual center-surround computations in the mouse superior colliculus

**DOI:** 10.1371/journal.pbio.3003414

**Published:** 2025-10-16

**Authors:** Peng Cui, Kuisong Song, Dimitrios Mariatos-Metaxas, Arturo G. Isla, Teresa Femenia, Iakovos Lazaridis, Konstantinos Meletis, Arvind Kumar, Andreas A. Kardamakis

**Affiliations:** 1 Department of Neuroscience, Karolinska Institutet, Stockholm, Sweden; 2 Instituto de Neurociencias (Consejo Superior de Investigaciones Científicas—Universidad Miguel Hernández), San Juan de Alicante, Alicante, Spain; 3 Tecnológico de Monterrey, Escuela de Medicina y Ciencias de la Salud, Monterrey, México; 4 Department of Neurobiology, Care Sciences and Society, Karolinska Institutet, Stockholm, Sweden; 5 McGovern Institute, Massachusetts Institute of Technology, Cambridge, Massachusetts, United States of America; 6 Division of Computational Science and Technology, School of Electrical Engineering and Computer Science, KTH Royal Institute of Technology, Stockholm, Sweden; 7 Science for Life Laboratory, Solna, Sweden; Yeshiva University Albert Einstein College of Medicine, UNITED STATES OF AMERICA

## Abstract

Models of visual salience detection rely on center–surround interactions, yet it remains unclear how these computations are distributed across retinal, cortical, and subcortical circuits due to their overlapping contributions. Here, we reveal a *de novo* collicular mechanism for surround suppression by combining patterned optogenetics with whole-cell recordings from individual neurons in the mouse superficial superior colliculus (SCs). Center zones were defined by monosynaptic input from channelrhodopsin-expressing retinal ganglion cells in collicular midbrain slices. Surround network optoactivation suppressed center responses compared to center-only input. This suppression is excitatory in origin, arising from the withdrawal of center excitation via surround-driven inhibition of local recurrent excitatory circuits, as demonstrated by cell-type-specific *trans-*synaptic tracing and computational modeling. These findings identify a local circuit mechanism for saliency computation in the SCs, independent of cortical input.

## Abstract 2

Visual salience generation involves center-surround dynamics, but which components of the visual system perform these computations as opposed to inheriting them from other regions? This study shows that the mouse superior colliculus encodes center-surround dynamics under isolated conditions, and provides insights into the circuit implementation.

## Introduction

Center–surround interactions drive neurons to respond optimally to stimuli within their central receptive field while being suppressed by competing input from their surrounding region. In the vertebrate visual system, surround suppression originates in the retina [1–4] and is observed across multiple stages, including the lateral geniculate nucleus [5], primary visual cortex (V1) [6–9], extrastriate cortex [10], and retinotectal structures such as the premammalian optic tectum [11–13] and its mammalian homologue, the superior colliculus (SCs) [14–16]. Because these processes are distributed across the visual hierarchy and most circuits—including the SC—integrate multiple upstream signals, it remains challenging to disentangle local computations from inherited input. Determining where computations arise *de novo* is key to revealing how visual saliency is generated and how feedforward and feedback influences are integrated across the visual system.

**Fig 1:**
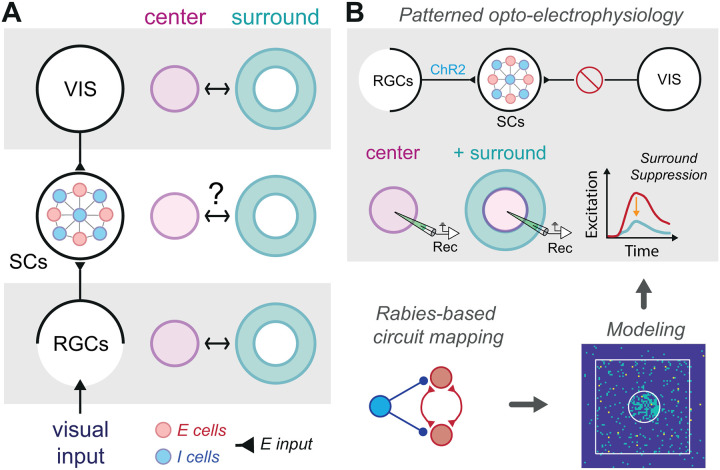
Experimental and computational approaches reveal circuit basis of collicular surround suppression. (A) *(Left)* Schematic illustrating the two primary sources of glutamatergic visual input (E input) targeting excitatory (E cells, red), and inhibitory neurons (I cells, blue) in the superficial layers of the superior colliculus (SCs), originating directly from retinal ganglion cells (RGCs) and indirectly from the visual cortex (VIS).*(Right)* Visual center and surround interactions are well understood in the retina and visual cortex. However, it remains unclear whether the observed surround modulation in the SCs [14,15] is simply conveyed from suppression already occurring in the retina and visual cortex, or if the SCs can actively generate center-surround interactions. **(B)** *(Top)* Schematic of the patterned optogenetic setup used to probe local circuits in the superficial superior colliculus (SCs). Channelrhodopsin (ChR2) is selectively expressed in retinal ganglion cells (RGCs) to drive presynaptic input to SCs neurons, while cortical input from visual cortex is surgically removed, indicated by the barred circle. Whole-cell recordings from individual SCs neurons measure responses to spatially defined optogenetic activation of center and surround zones. Representative excitatory conductance traces shown in Fig 2H, Top) show that surround stimulation reduces center responses compared to center-only activation, revealing robust surround suppression. *(Bottom left)* Cell-type-specific, rabies-based *trans-*synaptic tracing is used to map excitatory *E* and inhibitory *I* connectivity motifs in the SCs. *(Bottom right)* Anatomical connectivity data informs a large-scale computational model of SCs microcircuits. The model simulates spike responses to center and surround stimulation and is used to explore how local *E–E, I–E, E–I I–I* motifs implement surround suppression. An arrow linking the model to the physiological recordings above indicates that model results help interpret the circuit logic underlying the experimentally observed suppression.

Although visual saliency has been extensively studied in cortical circuits [17–20], its encoding in the SCs remains less well understood (Fig 1A). Notably, neural correlates of saliency have been observed in the primate superficial SCs [21–23]. Yet, despite substantial top-down input from visual cortex, it remains unclear whether the SC relies on cortical feedback to compute saliency or can generate it *de novo* through direct retinal input and intrinsic circuitry, such as the long-range inhibitory connections identified in mice [24]. If the SC is capable of computing center–surround interactions independently, then identifying the local mechanisms involved becomes essential—not only to understand how saliency is computed subcortically, but also how these computations might be reshaped by cortical input.

To investigate the collicular mechanisms of surround suppression, we isolated the SCs from upstream influences by triggering SCs activity through selective control of retinal ganglion cell (RGC) input (Fig 1B). Channelrhodopsin was expressed in RGCs, and center zones were defined by using whole-cell recordings to identify monosynaptic excitatory responses evoked in SCs neurons during patterned optogenetic activation of RGC axon terminals in midbrain slices. This approach enabled spatial mapping of center–surround domains. Optogenetic activation of these zones in a collision-style protocol revealed robust surround suppression in individual SCs neurons. Surround input reduced center excitation without increasing inhibition compared to center-only stimulation, echoing normalization mechanisms in cortex [25] and suggesting disruption of center recurrent excitatory circuits. *Trans*-synaptic mapping identified excitatory-to-excitatory (*E–E*) and inhibitory-to-excitatory (*I–E*) motifs within the SCs, both of which are recruited by RGC input, as further supported by in vivo cFos induction. Model simulations showed that surround-driven inhibition alone could not suppress center excitability without strong local *E–E* connectivity, underscoring the importance of recurrent excitation. These findings suggest that collicular surround suppression not only exists—supporting its potential role in visual salience detection—but also operates through circuit principles similar to those underlying suppression in recurrent cortical networks [26–29].

## Results

### Mapping center zones in single SCs neurons

**Fig 2:**
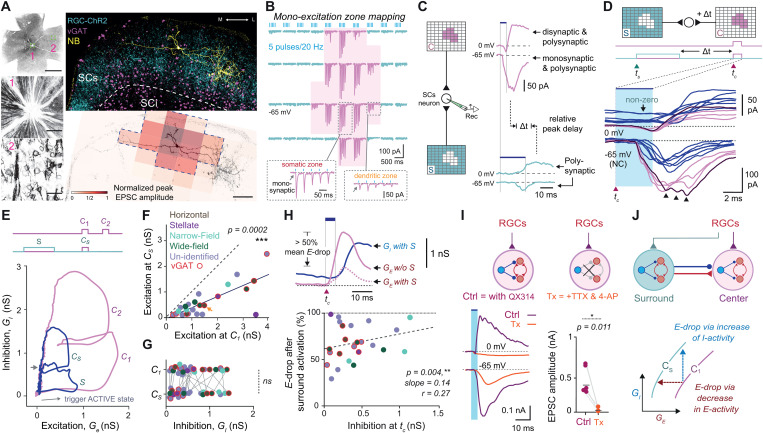
Visual surround network activation modulates center excitability in the SCs. ***(A)** *(Left)** From top to bottom: Inverted fluorescent image of whole retinal mount showing homogeneous transfection of RGCs with ChR2-EYFP. Scale bar: 1,200 μm. Images below show enlarged aspects of the optic disc (1) and individual RGCs (2). Scale bar: 25 μm. *(*Right*)* Top: A coronal section of the SCs that shows a representative example of this approach applied to an inhibitory horizontal SCs neuron (vGAT+, magenta) stained intracellularly with Neurobiotin (NB, yellow). ChR2-expressing RGC axonal terminals are shown in cyan. Bottom: Reconstructed neuron is shown with the denoted excitation zone (dotted line), which is referred to as center, whereas the non-responsive areas as surround (S) zone by mapping its monosynaptic excitation zone (shown in B). Scale bar: 100 μm. **(B)** Excitation zone mapping applied in neuron in (A) used for center zone determination. Light pulse trains (20 Hz, 5 ms) trigger excitatory postsynaptic currents (EPSCs, shown in magenta) with fixed short-latency responses suggesting monosynaptic transmission. Each trace is evoked from a single optostimulated rectangular area of 100 × 70 μm. When the rectangle is positioned over somatic zones, the responses are larger compared to those from dendritic zones; together, they delineate the overall center response zone of the neuron. ***(C)** *(Left)** Schematic showing two different light patterns used for optostimulation of ChR2-expressing RGC axonal terminals in the center (top) and surround (bottom) zones, while whole-cell recording postsynaptic responses from an SCs neuron. *(Top right)* Optostimulation of the center zone evoked excitatory and inhibitory postsynaptic currents when recording near reversal equilibriums for GABA_A_-chloride mediated inhibition and glutamatergic excitation (−65 and 0 mV, respectively). *(Bottom right)* Surround network activation triggers long-latency polysynaptic excitatory and inhibitory activity. The relative delay between peak center excitatory and peak surround inhibitory, *Δ*t**, was on average 20 ms. Traces were not corrected (NC) for the offset created by the liquid junction potential, which was experimentally confirmed to be ~ 10 mV. Throughout all experiments, light intensity was held constant for both the center and surround zones (<0.5 mWatt/mm^2^). **(D)** Synaptic modulation between the center and surround zone of the neuron shown in (A–C). Top: Surround and center interaction performed in a collision-style fashion by introducing delay *Δt* be*t*ween surround and center stimulation. (Bottom) Individual traces of synaptic inward and outward currents evoked in response to center stimulation, magenta/blue for center stimulation without/with surround stimulation, respectively. Arrowheads show oligosynaptic or recurrent activity in current traces with bold lines. Scale bar: 100 μm. Full traces in S2 Fig. **Abbreviation:* NC*, not corrected. ***(E)** *(Top)** Optostimulation patterns for paired-pulse center-only (C1-C2) and single-pulse transition surround-to-center activation (S-C_*s*_). *(Bottom)* Plot of the estimated inhibitory conductance as a function of the excitatory conductance in the neuron shown in (A–D). Arrow shows direction of process when neuron is activated. Smaller gray arrow indicates point of center stimulation in the surround-to-center case. **(F–G)** Comparison of estimated synaptic excitatory (F) and inhibitory (G) conductances between two conditions: center-only (C_1_) and center after surround (C_s_). Color coding corresponds to morphologically or genetically identified SCs neuron cell-types. Orange arrow depicts data point from the neuron shown in (A–D). ***(H)** *(Top)** Estimated excitatory conductance triggered by center-only (C_1_; solid red line) and center-surround (C_s_; dashed red line) condition. Inhibitory conductance (blue) in the center-surround condition; note surround inhibition is non-zero at time *t*_*c*_. *See* S3 Fig for all *t*races. *(Bottom)* Modulation of center excitation plotted as a function of surround inhibition at *t*_*c*_. **(I)** (Top and bottom left) Excitatory and inhibitory current recordings (at −65 and 0 mV) from SCs neurons (*n* = 5) were performed in response to center optostimulation of ChR2-expressing RGC axonal terminals. Bath application of 1 μM TTX and 100 μM 4-AP isolate monosynaptic RGC-triggered excitation while simultaneously abolishing recurrent excitation and recruited inhibition. (Bottom right) Statistical comparisons were performed as paired *t t*ests followed by Wilcoxon rank-signed test. ***(J)** *(Top)** Circuit diagram illustrating the direct and indirect actions of surround inhibition in the SCs through connections between excitatory (red) and inhibitory (blue) neurons in the center and surround. Note the recurrent connections between excitatory neurons. *(Bottom)* Schematic showing two theoretical lines indicating iso-conductance curves relating excitation to inhibition. Response suppression involves transitioning from a higher level to a lower level curve. The cartoon shows how the same level of response suppression can be achieved by either decrease in excitatory conductance or by increase in the inhibitory conductance. Statistical testing was done using *t* test followed by Wilcoxon rank-signed test (** *p* < 0.01, *** *p* < 0.001; ns, no significance). The data underlying this figure can be found in the Supplementary section.

First, we delineated the excitation zones of individual neurons in acute midbrain slices by identifying subregions within the SCs layer that received monosynaptic input from RGCs, enabling us to distinguish center and surround zones for selective stimulation and analysis of their interactions. To achieve this, we generated a light-excitable input field in the SCs that preserved the natural topographic organization of the retinotectal pathway [30] by intravitreally injecting an adeno-associated virus (AAV) expressing ChR2 into both wild-type (*n = 22, N = 14*) and vGAT-Cre (*n = 7, N = 5*) mice (Fig 2A, *see also* S1A Fig). Fig 2A and 2B illustrates how SCs center zones were identified (*n = 29, N = 19*) with a vGAT+ horizontal cell shown as a representative example. Short-latency EPSCs indicative of monosynaptic input (held at –65 mV) were evoked by patterned optogenetic stimulation (5 pulses at 20 Hz) across a 10 × 10 grid of 100 × 70 µm subregions. Subregions eliciting EPSC amplitudes above 10%–20% of the peak collectively formed a two-dimensional spatial profile used to define center boundaries relative to the surround, typically encompassing the cell soma and the majority of dendritic processes (S1C–S1F Fig).

Selective activation of the entire center zone consistently evoked robust short-latency compound EPSCs (120.8 ± 15.5 pA at –65 mV; magenta traces, Fig 2C), often with prominent multimodal peaks (arrowheads, Fig 2D), suggesting contributions from oligosynaptic and/or recurrent activity in addition to monosynaptic RGC input. Consistent with previous studies [11,24,31], we also observed di-/oligosynaptic IPSCs (24.3 ± 5.0 pA) near the glutamate reversal potential (at 0 mV; magenta traces, Fig 2C and 2D). In contrast, surround-evoked responses were long-latency and polysynaptic, requiring local interneuronal recruitment (cyan traces, Fig 2C). Latency analysis revealed that surround-evoked excitation and inhibition peaked at 20 and 28 ms, respectively, while center responses peaked earlier at 9 ms (excitation) and 15 ms (inhibition) (S3D Fig). Notably, pure inhibitory long-range responses from the surround zone were rare (e.g., S2B Fig); instead, both center and surround stimulation elicited mixtures of excitatory and inhibitory activity, differing in amplitude and timing (right panel of Fig 2C).

### Center-surround dynamics in brain slices of the superior colliculus

Center–surround interactions were subsequently induced in a collision-style manner by synchronizing stimulation to the average ~20 ms delay between peak center excitation and peak surround inhibition (Figs 2D, S3C, and S3D). Surround optostimulation was therefore terminated 20 ms before center onset (*t*_*c*_); moreover, it lasted 20 ms to allow sufficient buildup of surround-driven inhibition while minimizing overlap with faster excitatory components (see S2 Fig and Materials and methods for tested timing ranges). At *t*_*c*_, only inhibitory currents from the surround remained (see arrow in Fig 2D), as excitatory responses had already decayed due to their faster time constants and smaller amplitudes (blue traces, Fig 2D). During this interaction, peak center responses consistently declined in excitability, as evidenced by a >50% reduction in EPSC amplitude in this recorded neuron when surround stimulation was added (compare magenta versus blue traces, Fig 2D). Over half of the neurons were classified by morphology (i.e., horizontal, wide-field, narrow-field or stellate; *see* [32]) or genotype (e.g., vGAT+; S4 Fig), and based on the reduction in center excitatory conductance (Fig 2H), suppression was categorized as strong (>60%, *n* = 9/24), moderate (20%–60%, *n* = 10/24), or mild (<20%, **n* *= 5/24). Together, these results suggest that although the degree of suppression varies, surround-driven reductions in center responsiveness compared to center-only trials are a common feature among SCs neurons.

To determine whether surround suppression was driven by increases in levels of inhibition or decreases in level of excitation, we estimated excitatory (*G*_*e*_) and inhibitory (*G*_*i*_) conductances using a standard method [33,34] based on measured opto-evoked synaptic currents (*see* also Materials and methods). In the same neuron shown in Fig 2A–2D (see also S3A Fig), center–surround stimulation (S–C_s_, blue) led to a ~ 50% reduction in peak excitation compared to center-only stimulation (C1, magenta), without a corresponding increase in inhibition (Fig 2E). Notably, inhibition did not appear saturated: when center stimulation was repeated (C1–C2), the second pulse elicited nearly double the inhibitory response of the first, indicating that inhibition could still be recruited further. This rules out a ceiling effect and implies that surround suppression does not simply result from stronger inhibition. Rather, it suggests that surround activity may suppress center responses indirectly by diminishing excitatory drive within the SC circuit (*see* top panel in Fig 4A).

**Fig 3:**
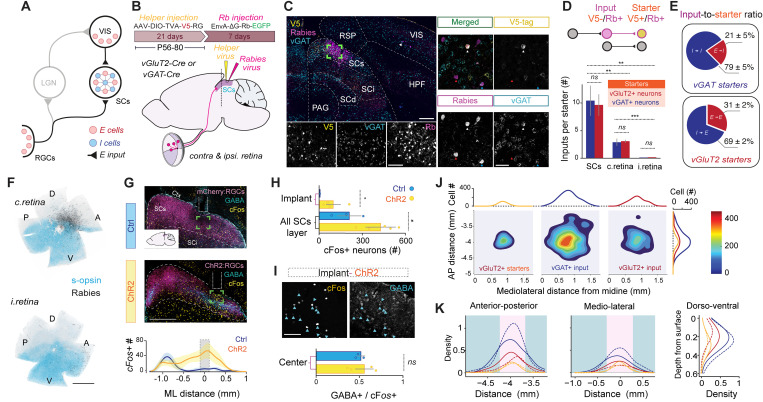
Connectivity logic between local excitatory and inhibitory neurons in SCs space. **(A)** Connectivity diagram of the early visual system highlighting the main sources of visual excitation arising from retinal ganglion cells (RGCs) and visual cortical (VIS) input targeting excitatory and inhibitory neurons in the SCs. **(B)** The helper virus AAV-DIO-TVA-V5-RG expresses the rabies glycoprotein (RG), and a fusion of the TVA receptor with the V5 tag. The RG-deleted rabies virus Rb, pseudotyped with EnvA, expresses EGFP. The AAV helper virus was first injected into vGluT2-Cre (*N = *3) and vGAT-Cre (*N = *3) mice and 3 weeks later the Rb-EGFP was delivered in the same location in the SCs. ***(C)** *(Left)** coronal section of the midbrain of a vGAT-Cre animal with emphasis on initial injection site where expression of the V5 tag (in yellow) should be restricted to and not beyond the SCs layer. Subsequently, EGFP-expressing neurons are a result of Rb-transfection (magenta). Scale bar: 300 μm. Insets below show colocalization of the enlarged area (green box). Scale bar: 100 μm. (*Right*) RNAscope in situ hybridization for *vGAT* (for *vGluT2*, *see* S5 Fig) revealed the identity of Rb-EGFP input neurons while confirming starter neuron cell-types. White arrows indicate starter neurons, whereas red and blue show excitatory and inhibitory input neurons, respectively. Scale bars: 50 μm. ***(D)** *(Top)** Diagram showing how starter neurons are identified based on the co-expression of the V5 tag and Rb-EGFP. Input neurons only express Rb-EGFP and provide monosynaptic and presynaptic input to the starter neurons. *(Bottom)* Input convergence shows the ratio of input-to-starter neurons, i.e., N(input)/N(starter), arising from: i) within the SCs, ii) contralateral (c.), and iii) ipsilateral (i.) retina. **(E)** Quantitative cell-type-specific input-to-starter ratios (referred to as input convergence) reveal feedforward (i.e., *E*–*I* and *I*–*E*) and recurrent connectivity patterns (i.e., *E*–*E* and *I*–*I*) calculated by n(vGAT+)/n(Total) and n(vGluT2+)/n(Total) presynaptic input neurons. **(F)** Contralateral and ipsilateral retinas showing retinotopy of presynaptic Rb-EGFP RGCs (black). Counterstaining with s-opsin (light blue) reveals retinal orientation. Dorsal, D; anterior, A; ventral, V; posterior, P. Scale bars: 1,000 μm. ***(G)** *(Top)** Coronal midbrain sections of the SCs centered around the implant for *cFos* induction following local optostimulation. Yellow, *cFos* immunostaining; magenta, control (top) or ChR2 (bottom); cyan, GABA immunostaining. Scale bar: 500 μm. *(Bottom)* The location and distribution of *cFos*+ neurons across the SCs. Blue line is control; Yellow line is ChR2-induced. The central zone (C) is defined by the implant tip, which defines the origin of the x-axis. Shading is standard error. **(H)** Comparison of the total cell count of *cFos*+ cells induced in SCs neurons between control (Ctrl, blue; *N = 3*) and during activation of ChR2-expressing RGC axonal terminals (ChR2, yellow; *N = 5*) across the entire SCs layer and center zone, respectively. Comparison of means performed using a *t* test. ***(I)** *(Top)** Grayscale image taken from the center region (*see* green rectangles in *G*) showing *cFos* (left) and GABA immunostaining (right). Blue arrows show colocalization. *(Bottom)* Ratio of GABA+ to *cFos*+ cells compared between the two conditions (ChR2, *N* = 4; and Ctrl, *N* = 3). Statistical testing was done using *t* test (* *p* < 0.05; *** *p* < 0.001; ns, no significance). Bars show standard error of the mean. ***(J)*** Contour plot showing the spatial distribution of *E* and *I* input neurons to *I* starter neurons in the AP and mediolateral (ML) dimensions across the SCs (for case shown in S5E Fig). **(K)** Quantitative relationship between the density of input neurons and starter neurons for *E* and *I* cell types across the three anatomical dimensions (AP, ML, and DV). Solid lines depict data obtained from excitatory starters averaged from vGluT2-Cre animals (*N* = 3), whereas dashed lines show data obtained from inhibitory starters averaged from vGAT-Cre animals (*N* = 3). See S5F Fig for the distributions obtained from individual animals. Magenta shaded area depicts starter zone; cyan-shaded area is outside the starter zone. *Abbreviations:* c., contralateral; i., ipsilateral; RGCs, retinal ganglion cells; LGN, lateral geniculate nucleus; VIS (and *V1*)*,* primary visual cortex; SCs, superior colliculus superficial layer; SCi, superior colliculus intermediate layer; SCd, superior colliculus deep layer; RSP, retrosplenial cortex; PAG, periaqueductal gray matter; HPF, hippocampal formation. The data underlying this figure can be found in the Supplementary section.

**Fig 4:**
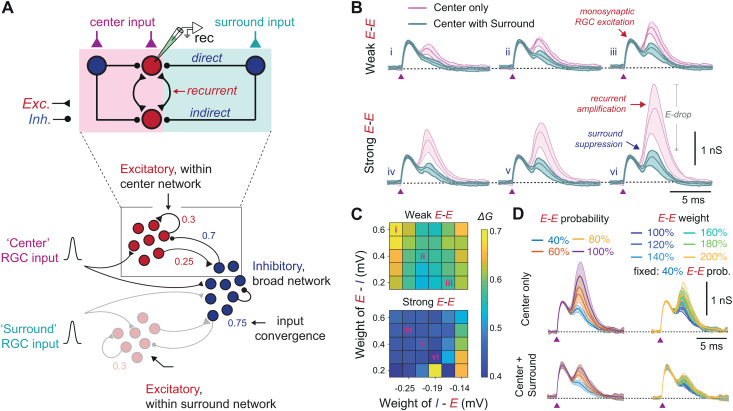
Modeling predicts that surround suppression depends on strong recurrent excitation. **(A)** *(Top)* A schematic breakdown of how surround stimulation suppresses the excitability of a center neuron (labeled "rec"), either directly via inhibitory input or indirectly by disrupting recurrent excitation. Direct inhibition increases inhibitory conductance onto the recorded neuron, whereas indirect suppression—through inhibition of excitatory partners—reduces excitation without elevating inhibition, appearing as a withdrawal of excitatory drive. *(Bottom)* Schematic of the *E*/*I* connectivity in SCs network highlighting the concept between center and surround in context of retinal input. Network model was simulated based on spatial distribution and input convergence using 6,400 excitatory and 6,400 inhibitory neurons. Raster of spiking activity and spatial arrangement of center and surround network response profiles are shown inS7 Fig. Initial parameters for connectivity probabilities and input convergence are obtained from circuit mapping in Fig 3 and then varied along with their synaptic weights to simulate their effect on surround suppression. **(B)** Time courses of excitatory conductance of a center neuron. Each trace is an average of 20 trials and shading indicated 25%–75% quartiles. The first peak is generated with low-variance monosynaptic activation of direct retinal input whereas the second peak is a network effect generated by balanced recurrent excitation and recurrent inhibition. The connectivity parameters for these six examples are marked in inset (C) with Latin numerals. **(C)** Normalized center conductance during surround stimulation. Each colormap shows normalized change in excitatory conductance (*see* Materials and methods) in a center neuron shown in (B) as a function of recurrent excitation (top: weak connection; 0.13 mV; bottom: strong connection; 0.26 mV) and mutual connectivity weights between excitation and inhibition (x-axis: inhibition of excitatory neurons; y-axis: excitation of inhibitory neurons). **(D)** Time courses of excitatory conductance in a center neuron under center-only stimulation (top traces) and center-plus-surround stimulation (bottom traces), shown under two conditions: (left) progressive decreases in *E–E* connection probability and (right) progressive increases in *E–E* synaptic weight at a fixed 40% recurrence probability.

Quantification revealed that peak center excitation (*G*_*e*_) was significantly reduced following surround activation (Fig 2F; C_1_: 1.7 ± 0.3 nS versus C_s_: 0.6 ± 0.1 nS; average decrease of 62.3 ± 4.7%, *p* = 0.0002). Similarly, the peak total conductance (*G*_*t*_ = *G*_*e*_ + *G*_*i*_) dropped from 2.1 ± 0.3 nS to 1.2 ± 0.2 nS, corresponding to a 40.6 ± 5.3% reduction (*p* = 0.0007; see S3B Fig). However, peak inhibitory conductance (*G*_*i*_) remained unchanged (Fig 2G; C_1_: 0.53 ± 0.08 nS versus C_s_: 0.55 ± 0.09 nS). Finally, bath application of gabazine (GBZ) abolished the surround-induced suppression, leading to saturated, burst-like center responses (S2A Fig, bottom), confirming the essential role of GABAA receptor–mediated inhibition in center–surround interactions.

### Direct and indirect inhibitory effects mediate surround suppression

Two mechanisms could underlie the observed reduction in center excitatory conductance: (i) direct surround inhibition—either linear or nonlinear—and (ii) indirect surround inhibition that suppresses activity from center excitatory neurons (Fig 2J, *see* schematic in Fig 4a, *Top*). Although a significant correlation was found, the shallow slope suggests that direct inhibition does contribute but cannot fully account for the observed reduction in center excitability (Fig 2H bottom). If direct inhibition were the primary driver of surround suppression, we would expect a steep, origin-centered relationship between surround inhibition (x-axis) and decrease in center excitation (y-axis). Instead, large excitatory reductions often occurred with minimal inhibition, suggesting additional contributing mechanisms.

An alternative mechanism involves suppression of excitatory inputs that amplify center responses. Under center-only stimulation, recurrent excitatory (*E–E*) connections enhance excitability beyond the initial monosynaptic RGC input. In this case, surround inhibition also acts indirectly by disrupting these excitatory loops, thereby reducing the overall excitatory drive. To probe this, we recorded from the center zones of SCs neurons (*n = 5*) during RGC terminal stimulation in the presence of TTX and 4-AP. EPSC amplitudes dropped sharply (control: 405.5 ± 81.1 pA; Tx: 22.3 ± 11.0 pA), indicating that polysynaptic excitatory inputs could contribute to center responses under normal conditions (bottom traces, Fig 2I bottom left), while the absence of outward currents at 0 mV confirmed the loss of recruited or feedback inhibition (top traces, Fig 2I bottom left). While these results support a potential role for polysynaptic excitation, we next turned to *trans-*synaptic mapping to directly map and visualize the excitatory and inhibitory connectivity motifs underlying this effect in the SCs.

### SCs space defined by recurrent excitatory and inhibitory circuit motifs

We used cell-type-specific retrograde circuit mapping to identify (*E*–*E*) connectivity and inhibitory motifs within the SCs could shed light on the collicular surround suppression observed in Fig 2. In contrast to previous studies emphasizing long-range input analysis [35], we mapped local connectivity patterns between vGluT2⁺ and vGAT⁺ neurons within the SCs and quantified their direct input from RGCs. To achieve this, we performed low-volume injections of a helper virus into the SCs of vGluT2-Cre and vGAT-Cre mice, enabling Cre-dependent expression of the TVA receptor and rabies glycoprotein (RG) in excitatory or inhibitory neurons, respectively (Fig 3B). Three weeks later, we injected an EnvA-coated, G-deleted rabies virus encoding EGFP into the same site (Fig 3B and 3C). ‘Starter’ cells were identified as those co-expressing EGFP and the V5 tag (via immunohistochemistry), while presynaptic ‘input’ neurons to the starter cells were labeled with EGFP alone (Fig 3C–3E). For example, in vGluT2-Cre mice (*N* = 3), starter neurons were excitatory; in vGAT-Cre mice (*N* = 3), they were inhibitory. Finally, to determine the neurotransmitter identity of input neurons, we performed RNAscope in situ hybridization targeting vGluT2 or vGAT mRNA (Figs 3C and S5), enabling cell-type-specific quantification of monosynaptic connections.

We quantified (1) the input convergence (as the number of input neurons per starter neuron) from three top sources (Fig 3D) and (2) the ratios of vGluT2+ or vGAT+ input neurons to each starter neuron subtype (Fig 3E). Local SCs inputs to neighboring vGluT2⁺ and vGAT⁺ starter neurons were comparable, averaging 9.5 ± 2.0 and 10.2 ± 2.8 cells, respectively, and represented the most prominent source of input (*N = 3* for vGluT2 and *N = 3* for vGAT starters). Moreover, the ratio of excitatory and inhibitory inputs to both starter cell types were similar. All four local connectivity motifs were present: vGluT2-to-vGluT2 (E–E), vGluT2-to-vGAT (E–I), vGAT-to-vGluT2 (I–E), and vGAT-to-vGAT (I–I) (Fig 3E). Inhibitory inputs were predominant for both inhibitory (78.8 ± 4.6%) and excitatory (69.2 ± 2.3%) starter neurons, with excitatory inputs making up the remaining 21.2 ± 4.6% and 30.8 ± 2.3%, respectively. The fact that nearly one-third of excitatory inputs specifically targeted excitatory neurons provides clear evidence for a recurrent excitatory network embedded within the densely intermingled SC circuitry.

Long-range inputs from contralateral and ipsilateral RGCs formed monosynaptic connections with both excitatory and inhibitory SCs neurons in balanced proportions (Fig 3D; contralateral: 2.9 ± 0.3 to vGluT2⁺ and 2.8 ± 0.7 to vGAT ⁺ ; ipsilateral: 0.7 ± 0.01 to vGluT2⁺ and 0.3 ± 0.02 to vGAT⁺), suggesting parallel engagement across these two cell types. RGC inputs followed a retinotopic organization, with contralateral RGCs originating from central retina and ipsilateral ones from peripheral regions (Fig 3F). To functionally assess this recruitment, we expressed ChR2 in RGCs, optogenetically stimulated the SCs in vivo, and used cFos and GABA immunostaining to label active inhibitory neurons (Figs 3G and S6). Optostimulation led to a marked increase in cFos expression beneath the implant in RGC-ChR2 mice compared to controls (Fig 3G and 3H), with almost half of the activated neurons in the center zone identified as GABAergic (Fig 3I). Excitatory (GABA^−^) and inhibitory (GABA⁺) neurons were recruited in comparable numbers, with a mean GABA+ /cFos+ ratio of 0.49 ± 0.07 (*N* = 4; Fig 3I). Although the longer timescales of cFos induction allow for possible indirect activation, such as via cortical feedback, the close correspondence with rabies-based input patterns supports the conclusion that retinal input engages both excitatory and inhibitory SCs neurons.

### Inhibitory input spans a wider spatial range than excitatory input

Fig 3J and 3K illustrates the spatial distributions of vGluT2⁺ starter neurons along with vGluT2⁺ and vGAT⁺ input neurons, mapped by detecting V5 and RV-EGFP expression and registering their positions within midbrain sections using the Allen Reference Atlas coordinate system. Gaussian kernel-based density plots in Fig 3J show the horizontal distribution of these populations across the mediolateral (ML) and anteroposterior (AP) axes (see also S5E Fig). In this vGluT2-Cre animal, excitatory starter neurons (left panel) were clustered within a confined retinotopic region of the SCs and received input from both a broader excitatory pool (right panel) and an even more spatially extensive inhibitory pool (middle panel). Input neurons consistently showed higher normalized densities than starters, as seen in Fig 3K, with solid lines indicating averaged spatial distributions for excitatory starters and dashed lines for inhibitory starters. Notably, input-to-starter inhibitory connections (*I–E* and *I–I*) spanned a wider area along all ML, AP, and dorsoventral (DV) axes compared to excitatory connections. These spatial patterns reveal a stable and symmetric organization of SC connectivity, with broad inhibitory networks coexisting alongside more localized excitatory networks across most of the collicular space.

### Modeling predicts that surround suppression depends on strong recurrent excitation

Recurrent excitatory connections, supported by electrophysiology (Fig 2) and mapping (Fig 3), were incorporated into a large-scale network model to evaluate their contribution—alongside inhibitory motifs—to collicular surround suppression. The model consisted of 12,800 leaky integrate-and-fire neurons with a 1:1 ratio of excitatory and inhibitory cells, arranged into a center zone (314 *E* and 314 *I* neurons) and a surround zone (3,286 *E* and *3*,286 *I* neurons), each sampled from separate 80 × 80 grid for excitatory and inhibitory model neurons (*see* S7C Fig). In line with experimental observations (Fig 3), connection probabilities for E → E, E → I, I → E, and I → I were implemented with distance-dependent rules (Fig 4A; see S6 Table) along with broader inhibitory projections. While grounded in measured connectivity patterns, the model primarily tested how weak (0.13 mV) versus strong (0.26 mV) *E–E* synapses shape surround suppression. Detailed model parameters are listed in S3–S7 Tables.

To simulate center activation, we stimulated 314 excitatory and 314 inhibitory neurons and measured the evoked excitatory synaptic conductance in a randomly selected center neuron (Figs 4B and S7). Activation of the center alone (magenta traces) generated a biphasic response: a first peak driven by monosynaptic RGC input, followed by a second peak reflecting recurrent excitatory input from neighboring neurons. This second peak, however, was shaped by the strength of *E–E* connections and modulated by *I–E* feedback inhibition, which requires activation of local inhibitory neurons recruited by center-driven RGC excitation (Fig 4B and 4C; *see* also Fig 4A, top).

To assess the effect of the surround, we additionally stimulated 3,286 excitatory and 3,286 inhibitory neurons (Figs 4B and S7C). Co-activation of center and surround (cyan traces) consistently attenuated the second excitatory peak, primarily due to inhibitory feedback via *I–E* connections. As expected, stronger *I–E* coupling reduced the number of neurons crossing threshold, suppressing the secondary response through both direct inhibition and indirect suppression of recurrent excitation (Fig 4A, top). To quantify this effect, responses during surround stimulation were normalized to those evoked by center-only input. Suppression scaled with *I–E* strength, while *E–I* and *I–I* connections had minimal impact (Fig 4C). Notably, increasing *E–E* connectivity enhanced suppression—not by counteracting inhibition, but by amplifying center responses and rendering them more susceptible to surround-driven inhibition.

To test the importance of *E–E* connection number, we reduced it up to 40% of baseline (Fig 4D, left) and systematically increased the strength of the remaining synapses up to twice their original value (Fig 4D, right) using the same connection weights for *E–I*, *I–E*, and *I–I* as in condition “v” of Fig 4B. Reducing *E–E* connectivity to 40% of baseline lowered the secondary excitatory peak in both center-only and center–surround conditions, eliminating up to 47% of the secondary response (Fig 4D, left). Increasing the strength of the remaining *E–E* synapses—up to double the original weight—only partially rescued this loss, recovering just up to 69% of the baseline peak (Fig 4D, right). These results indicate that increasing *E–E* synaptic strength cannot fully compensate for reduced connectivity, and that robust recurrent excitation is not merely compatible with surround suppression but necessary for its expression.

## Discussion

Targeted optogenetic activation of center and surround zones in single neurons revealed the presence of a *de novo* surround suppression in the SCs, evidenced by reduced center excitation during surround network activation compared to center-only stimulation (Fig 2). This suppression has an excitatory basis, arising not from increased inhibition but from a withdrawal of excitation—due to a disruption of local recurrent circuits, as supported by modeling, in which surround-driven inhibition silences both the center neuron and neighboring excitatory partners that would otherwise amplify its response beyond monosynaptic RGC input. Without such recurrent amplification, suppression would require stronger direct surround inhibition—something not supported by our data. Fig 2J illustrates this process as a shift along excitation–inhibition iso-conductance curves, with our data supporting a transition primarily driven by reduced excitation (red dotted line in Fig 2J). This mechanism is supported experimentally and through modeling, which shows that suppression fails in the absence of strong E–E connectivity. Together, these findings define a circuit logic that is consistent with cortical models of visual suppression via recurrent amplification [26,28,36]. That such a mechanism operates in the SC—a structure predating the neocortex—suggests it may represent an evolutionarily conserved strategy for contextual visual processing.

Like cortical circuits [37], the SC contains densely intermingled excitatory and inhibitory neurons [38] (Fig 3A), complicating the analysis of their specific functional roles. By integrating data from whole-cell recordings (Fig 2), connectivity mapping (Fig 3), and computational modeling (Fig 4), we identified two core local motifs—recurrent excitation (*E–E*) and feedback inhibition (*I–E*)—that interact to shape collicular surround suppression. *E–E* connections selectively amplify activity within a retinotopically defined center, while *I–E* circuits provide recruited feedback that modulates both the center and extends laterally to suppress the surrounding network. While our analysis focuses on vGluT2⁺ (*E* neurons) and vGAT⁺ (*I* neurons) cells, it remains possible that excitatory and inhibitory inputs preferentially target specific SC cell types, such as wide-field, narrow-field, stellate, or other subtypes of SCs neurons, each of which are recipient to distinct retinal signals. Future work should refine this framework by moving beyond the broad *E* and *I* classes examined here and identify how specific SC cell types contribute to these connectivity motifs.

To explore how these local circuit motifs contribute to network-level computations, we modeled the SCs using connectivity ratios and probabilities derived from transsynaptic mapping (*see* Fig 3). Our approach captures cell-type-specific input patterns to excitatory and inhibitory neurons; however, it may underestimate *E*/*I* ratios and does not convey synaptic strength. By embedding these experimentally derived values into a reduced yet informative large-scale model, we tested how connection density, probability, and synaptic weight shape center-surround interactions. The stimulation protocols were designed as a mechanistic test of the capacity for surround suppression in the SCs, yet they may also reflect physiologically relevant scenarios, as looming or small moving stimuli can activate peripheral SCs regions before medial ones -or vice versa- generating temporal dynamics similar to those modeled here. The results reveal a key principle: effective feedback inhibition (*I–E*) requires sufficiently dense recurrent *E–E* connectivity, as surround suppression depends on prior amplification of center activity by excitatory loops. Notably, sparse *E–E* connectivity cannot be compensated by stronger synapses, underscoring that approximate *E*/*I* ratios are sufficient to capture the core interaction. This suggests a hierarchical organization in which excitation enables inhibition, rather than opposes it. Finally, models of trial-by-trial variability suggest that, while both increases in inhibition or decreases in recurrent excitation can similarly affect the average output response suppression, the latter is more likely to minimize variability across trials, thereby offering greater stability [39] than inhibition-dominated mechanisms [40].

Conducted in acute midbrain slices—functionally isolated from long-range inputs—these experiments point to a local *de novo* origin of retinal-dependent surround suppression within SC circuits. This helps clarify the relative contributions of retinal and cortical input to SC function, which is critical for understanding how bottom-up and top-down signals interact during saliency processing [17–20] and visuospatial attention [41–44]. In this context, the SCs provides upstream cortical circuits with the flexibility to modulate its activity by boosting or suppressing center-surround processes with retinotopic correspondence [41]. This organization may enable top-down inputs from areas like V1 to engage SC recurrent networks and help coordinate collicular suppression, hence visual salience detection. Applying the excitation-zone mapping approach introduced here to cortico-collicular pathways could also determine whether, for example, V1 pyramidal neurons selectively enhance SCs activity by accessing *E–E* connections or suppress it through *I–E* pathways. We anticipate that such studies will bring us a step closer to understanding how conserved subcortical circuits and neocortical pathways coordinate, cooperate, or compete—shedding light on the integrative circuit logic underlying visual salience.

## Materials and methods

### Ethics statement

Experimental procedures were approved by the Stockholm municipal committee for animal experiments and the Karolinska Institute in Sweden (N9179-2017 and N6747-2019 to A.A.K.), as well as, the Committee on Animal Research at the Universidad Miguel Hernández and carried out in compliance with the Research Council of Spain (CSIC), the Generalitat Valenciana (GVA) and European regulations (2022/VSC/PEA/0236 to A.A.K.).

### Animal strains

Both male and female vGAT-Cre (Jackson: Slc32a1tm2(cre)Lowl), vGluT2-Cre (Jackson: Slc17a6tm2(cre)Lowl), and wild-type mice (C57BL/6N; Charles River) were used throughout the experiments. Mice were kept on a 12-h day/night cycle under stable temperature (21 ± 1°C) and air humidity (50%–65%) with freely available food and water. The exact numbers of cells (*n*) and animals (*N*) used for each experiment are reported in the Results section and the corresponding figure legend.

### Viral injections

The complete list of viruses we used is provided in S1 Table. Injections were performed in 2- to 5-month-old wild-type mice and transgenic mice under isoflurane anesthesia and injected with buprenorphine post-surgery. Viral titers of AAV and modified rabies virus were between 1 × 10^12^ and 1 × 10^13^ viral genomes per milliliter (vg/mL). To express ChR2 in the axons of RGCs in the superficial layer of the right hemisphere SC, we injected 1,000 nL into the vitreous bodies of the left retinas using the following viral vectors:

1. rAAV2/hsyn-hChR2(H134R)-EYFP-WPRE-PA (UNC Vector Core),
2. rAAV2/hsyn-hChR2(H134R)-mCherry-WPRE-PA (UNC Vector Core),
3. rAAV2-EF1α-DIO-hChR2(H134R)-EYFP (UNC Vector Core), or
4. ssAAV-2/2-shortCAG-dlox-hChR2(H134R)_EYFP(rev)-dlox-WPRE-hGHp(A) (VVF, Zurich).

We used the vector rAAV2/hsyn-mCherry (UNC Vector Core) as in our control group for the intravitreal injections. For trial inclusion, we first confirmed sufficient expression of ChR2 across the SCs and homogeneous pan-retinal expression of ChR2 (Fig 2A, Left); partial expressions were not taken into consideration to minimize the risk of false positive surround classification that could arise from lack of ChR2 expression. Only animals exhibiting uniform RGC expression across the retina, full optic disc coverage, and homogeneous fluorescence throughout the SCs were included in our analysis following quality control screening.

To identify GABAergic neurons in the SCs, the vector rAAV5-CAG-FLEX-tdTomato (400 nL, UNC Vector Core) was injected into two collicular sites in order to maximize expression: (1) the rostral SCs (from bregma: AP −3.6 mm; right (R) −0.8 mm; DV −1.6 mm from the surface of the skull), and (2) the caudal SCs (AP −4.2 mm; R −1.8 mm; DV −1.5 mm), respectively, on the same day.

For the rabies virus tracing, 50 nL of helper virus AAV5-EF1a-DIO-TVA-V5-t2A-RG was injected into the right SCs (AP −3.9 mm; R −0.8 mm; DV −1.05 to −1.15 mm from the surface of the brain) in vGAT-Cre or vGluT2-Cre mice, while taking great care to maintain viral spread to within the SCs layer. Three weeks later, 150 nL of the engineered rabies virus EnvA-coated G-deleted rabies virus with EGFP (EnvA-ΔG-Rb-EGFP) was injected into the same location. The low injection volume of the helper virus (50 nL, 1 × 10^12^ vg/mL) allowed for localized expression of the helper virus within SCs boundaries, which ensured minimal spillover to the intermediate layer (SCi). This also maintained a low infection rate (19% for vGAT+ and 47% for vGluT2+ ; *see* S5G Fig, top) allowing for sparse labeling of starter neurons, thereby increasing confidence in quantifying true input neurons (V5−/Rb+) within the starter zone, defined by the area containing co-labeled V5+/Rb+ starter neurons. The similar input expression probabilities inside and outside the starter zone (80% and 78% for vGAT+ and 65% and 66% for vGluT2+, respectively; *see* S5G Fig, bottom) further validated an accurate assessment of input neurons within the starter zone. Importantly, this similarity not only served as a control for reliable labeling but may also reflect a biological feature, which was incorporated into our modeling in Fig 4.

For secondary injections, fiber optic implantations and electrophysiological recordings, 2–3 weeks were allowed to pass after the initial injection. Viral suspensions were delivered at 500 nL/min and 100 nL/min for intravitreal and brain injections, respectively. A puncture was made 2 mm posterior to the corneal limbus using a sterile 30-gauge (G) needle after pupil dilation was achieved with 0.5% Alcaine. After which 1,000 nL vitreous was aspirated manually using a Hamilton microliter syringe. The glass capillary was then inserted through the same puncture by using a microliter syringe (1.5 mm volume) into the eye, and the tip was angled toward the vitreous humor and back of the eye, taking care to avoid any damage to the retina or lens. The capillary remained in the eye for 2 min in order to prevent reflux of the viral suspensions. Brain injections were performed by using a digital stereotaxic frame combined with a Quintessential Stereotaxic injector (Stoelting) via a glass capillary. The capillary firstly reached 0.2 mm deeper than the target location to give space for the viral suspensions, then was slowly retracted to the target location to complete the injections. Afterwards, the capillary was retracted by 0.2 mm again and remained in position for 8 min. During the injections, all the mice were kept on a heating pad at 37°C with a subcutaneous injection of buprenorphine while maintaining the eyes were moisturized with Viscotears gel.

### Patch electrophysiology in acute midbrain slices

The animals were deeply anaesthetized by using a combination of pentobarbital injected intraperitoneally. To ensure rapid cooling of the midbrain, we perfused the animals transcardially with an ice-cold cutting solution, containing the following (in mM): NaCl 40, KCl 2.5, NaH_2_PO_4_ 1.25, NaHCO_3_ 26, glucose 20, sucrose 37.5, HEPES 20, NMDG 46.5, Na L-ascorbic acid 5, CaCl_2_ 0.5, and MgCl_2_ 5 (pH 7.3 with HCl). The brain was rapidly dissected out after decapitation. Coronal midbrain slices (400 μm) were prepared by using a vibratome (VT1000S, Leica, Germany) in the cutting solution and incubated in the same solution at 35°C for 12 min. Slice orientation was chosen to profile responses along the medio-lateral axis corresponding to the spatial axis of the retina across the dorso-ventral direction of visual space, whereas the dorsal-ventral axis of the SCs slice corresponded to the various sublaminae of the SCs. The sections were subsequently maintained at room temperature in recording solution that contained the following (in mM): NaCl 124, KCl 2.5, NaH_2_PO_4_ 1.25, NaHCO_3_ 26, glucose 20, CaCl_2_ 2, and MgCl_2_ 1 (pH 7.3). Cutting and recording solutions were continuously infused with carbogen (95% O_2_ and 5% CO_2_) throughout the procedure.

Whole-cell patch clamp recordings were either performed on random neurons in the SCs of wild-type mice or tdTomato-expressing neurons of vGAT-Cre mice at room temperature using pCLAMP 11 (Molecular Devices, USA). Midbrain slices were continuously perfused with oxygenated recording solution at a rate of 2 mL/min during recording and visualized under an upright CleverScope motorized microscope (Micro Control Instruments, UK) equipped with a Dodt gradient contrast system (DGC-2), an oil condenser (U-AAC, Olympus, Japan), and two Chameleon3 cameras (Teledyne FLIR, USA). Whole-cell recordings were obtained under visual control using a 16× (0.80 NA) water dipping objective (CFI75 LWD 16X W, Nikon, Japan) combined with 2× c-mount extender (EX2C, Computar, USA) and c-mount adapters (U-TV1X-2 and U-CMAD3, Olympus, Japan) with Axon MultiClamp 700B amplifier and Axon Digidata 1320A digitizer (Molecular Devices, USA). Signals were sampled at 20 kHz. Pipettes used for recording were pulled from borosilicate glass capillaries (article no. 1403542, Hilgenberg, Germany) by a P-97 Micropipette Puller (Sutter Instrument, USA). Patch pipettes (5–8 MΩ) were filled with a potassium gluconate-based intracellular solution contained (in mM): K d-gluconate 125, EGTA 1, KCl 10, HEPES 10, ATP-Mg 4, GTP-Na 0.3, phosphocreatine 10, CaCl_2_ 0.1, and QX314 3 (pH 7.2 with KOH; 290 mOsm). Due to the long recording times (usually 1 h), the access resistance was evaluated every 10 min to ensure that initial values (only under ~30 MΩ were considered) did not increase throughout the recording session.

ChR2-expressing axons of RGCs were optically stimulated with 470 nm light from a high-power collimated LED source (Mightex, Canada) connected to a digital mirror device (DMD) (Polygon1000, Mightex, Canada) by a 3 mm-core liquid lightguide (Newport, USA). The stimulation patterns were triggered via Master-8 pulse stimulator (A.M.P.I, Israel) and designed via the software PolyScan2 (Mightex, Canada). The tdTomato-expressing vGAT-positive neurons in the SCs were detected with 540 nm light from a high-power collimated LED source (Mightex, Canada) and an RFP filter set (Olympus, Japan). The 540 nm LED was connected to a 495 nm dichroic filter cube (Mightex, Canada) with the 470 nm LED during the transgenic animal experiments. Both LEDs were directed via a four channel BioLED light source control module (Mightex, Canada).

For membrane property measurements, a series of 1,000 ms negative and positive injected current steps from −30 pA to +40 pA in 5 pA increments were delivered through the recording electrode under current-clamped conditions to each recorded neuron.

### Excitatory zone mapping

To accurately cover each neuron's center zone and ensure a significant surround area, we created a sufficiently large field of view (FOV) that could accommodate their distinct morphologies. We achieved this using one-photon widefield optics with a low magnification objective (16×) and an in-house built dual-focus visual inspection system that allowed selective magnification and demagnification of the optical path (Fig 2B). Demagnification (by 0.5×) captured a large FOV of >1 mm², encompassing most of the SCs (magenta box in lower right of S1A Fig), enabling patterned micro-optostimulation of the entire visual layer. The alternate path for magnification (by 2×) provided sufficient single-cell resolution to visually target neurons of interest for whole-cell recordings.

We combined whole-cell electrophysiology with a DMD-based dual-camera system to map the receptive fields of individual neurons. Optogenetic stimulation patterns were created and implemented during recordings by the DMD (Polygon1000, Mightex, Canada). The 16× objective, along with 2× front tube optics (Mightex, Canada) and a 0.5× single photo port tube lens camera adapter (Olympus, Japan), enabled a workable field-of-view of approximately 1.2 × 1 mm. Using this customized visual inspection system, whole-cell patch-clamp recordings were performed through the high magnification pathway while optostimulation patterns were executed via the low magnification pathway. All neurons were voltage-clamped to −65 and 0 mV to measure excitatory postsynaptic currents (EPSCs) and inhibitory postsynaptic currents (IPSCs), respectively, with an intermediate potential (−45 mV) applied for membrane potential recordings in most neurons when stable. Traces were not corrected (NC) for the offset created by the liquid junction potential, which was experimentally confirmed to be ~10 mV.

For control experiments, whole-field stimulation patterns were delivered by 10 Hz trains of 8 stimuli (5 ms duration), followed by a single test stimulus at 5 s time interval, and each trace included four repetitive sweeps. For the center-surround experiments, SCs neurons were subjected to the following patterns: “GridScan,” “Center-Center,”, “Surround-Surround,” and “Surround-Center.” Center (C) and Surround (S) areas were determined by using the GridScan protocol, which was delivered 10 Hz trains of 5 light pulses of 5 ms duration in 100 non-adjacent grids arranged in a 10 × 10 spatial configuration across the FOV (0.1 × 0.07 mm for each one). After the end of the GridScan, we promptly calculated the maximum EPSC and IPSC amplitudes for each grid after it was normalized and filtered using custom-written MATLAB (MathWorks). EPSC amplitudes arising from each grid location were thresholded on the basis of their evoked amplitude. Grid locations that yielded ≥10%–20% of the peak EPSC amplitude for the recorded neuron, were considered to be part of the center zone, whereas locations that did not pass this threshold were considered to be part of the surround zone. To trigger center-surround interactions, the surround was optostimulated by a single pulse that was then followed by a two or five-pulse train stimulation delivered to the center. We experimented with a range of different surround stimulation durations (5, 20, 50 ms) and with a range of surround-center delays (0, 10, 20, 50 ms) to explore the parameter space. The inter-trial interval was 25 ± 5 s.

### Drugs used in electrophysiology

Drugs were bath applied and an overall duration of at least 5 min was allowed before testing their effects, which was followed by a washout phase of at least 10 min. Unless otherwise indicated, drugs were perfused during patch-clamp recordings under the given conditions using a two-gauge peristaltic pump (Pretech Instruments, Sweden). The complete list of drugs used is provided in S2 Table.

### Estimation of synaptic conductances

To deconvolve inhibitory and excitatory synaptic conductance, recordings were performed during “center-center,” “surround-surround,” “surround-center,” and “global” optostimulation. A QX314-based intracellular solution was used in the recording pipette to improve intrinsic conductance isolation and block action potential discharge conditions (Figs 2, S1, and S2). Evoked synaptic currents used in this analysis were obtained by averaging values from 5 to 10 sweeps for each recorded cell. Due to the long recording times (usually one hour), the access resistance was evaluated every 10 min to ensure that initial values (only < ~30 MΩ were considered) did not increase throughout the recording session at each membrane potential. Customized MATLAB scripts were written to estimate the total, excitatory and inhibitory synaptic conductances based on the equations used in [33,34]. It should be noted that the postsynaptic currents were adjusted for the liquid junction potential (~10 mV) and taken into consideration when estimating for their underlying evoked synaptic conductance.

### Neuronal reconstruction

To identify the dendritic fields from the recorded neurons, we used 0.3% Neurobiotin (Vector Laboratories, no. VESP-1120-–50) in the intracellular solution during whole-cell recordings. We post-fixed the midbrain slices and then used PBS to wash them. To visualize the neurons, we stained them with streptavidin Cy5 (1:400, Jackson ImmunoResearch, no. 016-170-084) and imaged them using confocal microscopy with a 20× objective (LSM-800, Zeiss, Germany). The images were processed with ImageJ software, and the neurons were reconstructed by using the plugin Simple Neurite tracer. The dendritic areas of each labeled neuron were calculated by tracing a convex polygon around the outermost tips of the dendritic field. The SC cell types were then classified on the basis of their geometry [32].

### Fiber optic implantation and *cFos* activation

Two weeks after the intravitreal injection, a 200 μm diameter fiber optic (Thorlabs, FG200UEA) was implanted into the right SCs (from bregma: AP −3.6 mm; R −1.0 mm; DV −1.0 mm from the brain surface). The mice recovered for at least one week before the *cFos* induction experiment. The mice were habituated to handling in an open field area (60 cm × 60 cm × 60 cm; W × D × H) and the connected optic fiber for at least 3 days before proceeding with the experiment. To minimize stress, bedding from the cage was placed in the box. Optogenetic stimulation was not initiated for 90 min after the mice were placed in the dark box to avoid any prior visual effects on the observed *cFos* expression. The RGC axonal terminals in the SCs were stimulated with a train of 1 s square pulses of 40 Hz frequency (10 ms ON and 15 ms OFF) every 10 s for a total of 40 min. After the stimulation period, the mice were left undisturbed for 60–70 min to ensure sufficient *cFos* protein expression. A maximum of 90 min after the end of the stimulation period was allowed before the mice were euthanized with pentobarbital and perfused with ice-cold 4% paraformaldehyde (PFA) in 0.01 M (1×) phosphate-buffered saline (PBS) buffer. The brain and retinas were subsequently dissected, and post-fixed in 4% PFA overnight at 4°C.

### Immunofluorescent staining and in situ hybridization

Mice were anesthetized by pentobarbital and perfused with 1× PBS and 4% PFA. The brains (and eyes, to confirm pan-retina transfection) were harvested and fixed in PFA at 4°C overnight, then washed with PBS for three times. The brain was placed in 15% sucrose solution for 24 h, then transferred into 30% sucrose solution for 24–72 h. The brains were subsequently embedded in optimal cutting temperature compound, frozen with dry ice and kept at −20°C for short-term storage. The cryostat sectioning was performed on a Epredia NX70 cryostat. The brains, between the thalamus and brainstem, were sectioned in 14 μm thickness, about 80% sections in the SCs were collected on glass slides, and every 6th section with an interval of ~0.105 mm was sampled for subsequent staining (total of 12–15 sections per animal). The other parts of the brain were sectioned in 40 μm thickness and stored in 1 × PBS with 0.01% sodium azide.

The fluorescence in situ hybridization was performed with RNAscope Multiplex Fluorescent Reagent Kit v2 (ACD, Cat. No. 323100). The probes for *vGluT2* (Mm-Slc17a6-C2, ACD, Cat. No. 319171-C2) and *vGAT* (Mm-Slc32a1, ACD, Cat. No. 319191) were used to identify the cell types. The manufacturer’s protocol was followed. Co-staining of *vGAT* and *vGluT2* in situ hybridizations was also performed for control experiment, and the results were shown in S5 Fig. Specifically, co-staining of Rb-EGFP+ cells with *vGluT2* and *vGAT* probes revealed no colocalization, as neurons negative for *vGAT* were positive for *vGluT2* and vice versa, with nearly all detected input cells labeled by only one of the two probes (269 out of 272 in *N* = 2 control animals; S5A Fig).

Immediately following the in situ hybridization, the brain sections were incubated in chicken anti-V5 primary antibody (Abcam, ab9113) in 1:750 at room temperature overnight, then washed with 1× TBST (0.3% TritonX-100), and incubated in donkey anti-chicken Cy3 secondary antibody (Jackson ImmunoResearch, no. 703-165-155) in 1:1,000. All the antibodies were diluted with 1× TBST (0.3% TritonX-100, 3% BSA, 0.01% sodium azide). After PBS washing and DAPI staining, the sections were mounted with an antifade mounting medium (VWR, BSBT AR1109). For the *cFos* staining, rat or rabbit anti-*cFos* primary antibodies (Synaptic Systems, no. 226−017 or no. 226−003, 1:500) and GABA primary antibody (Sigma-Aldrich, A2052, 1:2,000) were used in 1× PBST (0.3% TritonX-100, 5% donkey serum, 0.01% sodium azide) at room temperature overnight, then the sections were stained with Cy3 donkey anti-rabbit (Jackson ImmunoResearch, no. 711-165-152) or AF647 donkey anti-rat (Jackson ImmunoResearch, no. 712-605-153). The same following steps are previously described.

The retinas were first removed from the eye in the perfused animals and then flattened with the help of four incisions, and finally washed in 1× PBS for three times. For the retina immunostaining, the retina was first treated with pre-heated (about 70°C) 1× antigen retrieval citrate buffer (Sigma, C9999), then followed the same protocol as with the brain section described above. Rabbit anti-S-opsin primary antibody (Sigma, AB5407) and donkey anti-rabbit Cy3 secondary antibody were both used in 1:1,000 to visualize the short-wave photoreceptors, which reveals retinal orientation.

### Network model

We used an integrate-and-fire model to simulate the dynamics of neurons. The subthreshold membrane potenn:


$$C_{m}dV_{m}/dt\;=\;g_{L}\;(V_{m}\;-\;E_{L})\;+\;{I^{bkg}}_{syn}\;+\;{I^{net}}_{syn}\;+\;{I^{stim}}_{syn},$$

where *C*_*m*_ is the membrane capacitance, *g*_*L*_ is the leak conductance, $I_{syn}^{net}$ is the total synaptic input from neurons within the network, $I_{syn}^{bkg}$ is the background input from other networks, $I_{syn}^{stim}$ is input corresponding to center or surround inputs (*see* S3 Table). When the membrane potential reached *V*_*th*_, spike was elicited and the *V*_*m*_ was reset to *E*_*L*_ for *t*_*ref*_ millisecond. In the network, all neurons were identical. Neuron parameters are provided in S4 Table.

Synaptic inputs were modeled as conductance transients with the following dynamics:


$$g_{syn}\;=\;J_{syn}t/{tau}_{syn}\;exp(-(t-(t_{spk}+\;d_{syn}))/tau)\;when\;t-t_{spk}\;>\text{0}\;else\;g_{syn}\;=\;\text{0},$$

where *t*_*spk*_ is the spike time, *d*_*syn*_ is the synaptic delay, *J*_*syn*_ *{syn: ee, ei, i.e., ii, ex, ix)* is the amplitude of the postsynaptic potential, and *tau*_*syn*_ is the synaptic time constant. In our model, all synapses of a particular type (external, excitatory, or inhibitory) have the same parameters (*see* S5 Table). In our model, we fixed the amplitude (*J*_*syn*_) of the external excitatory (*J*_*ex*_), inhibitory (*J*_*ix*_) synapses, and *J*_*ii*_ (inh. → inh.). The value of *J*_*ee*_ (exc. → exc.), *J*_*ei*_ (exc. → inh.), and *J*_*ie*_ *(inh. → exc.) were systematically varied. Synapse parameters are provided in* S5 Table*.*

We considered a network with 6,400 excitatory and 6,400 inhibitory neurons. Neurons were placed on an 80 × 80 grid. Because we have the same population size for the two neuron types, both types of neurons were placed on the same-size grid. Neurons were connected based on their spatial distance. To avoid boundary effects, the 80 × 80 grid was folded as a torus. The connection probability decreased as a function of distance according to a Gaussian function. The standard deviation of the Gaussian was set to 16 and 20 for excitatory and inhibitory synapses, respectively. This was based on our experimental data which showed that inhibitory neurons have a slightly broader extent of their spatial connectivity than that of excitatory synapses (Figs 3 and S5 Fig). Sixteen and twenty grid points constitute 2% and 2.5% of our network space. The area of the Gaussian function (*i.e.,* the total number of connections sent out by a neuron) was decided based on the input convergence we measured in our experiments (Fig 3K). Network connectivity parameters are provided in S6 Table.

Each neuron received Poisson-type excitatory and inhibitory spiking inputs to mimic inputs from outside the network and to obtain a low firing rate (<1 Hz) background activity. *See* S7 Table for parameters.

To model a short flash-like input to the center region, we selected 314 excitatory and 314 inhibitory neurons, located in a circle (radius = 10 grid points) at the center of our network model. These neurons received a single synchronous spike from 10 presynaptic neurons (from the retina). Each spike elicited 1.1 mV depolarization. Each neuron received such a synchronous spike event at the stimulus onset (*Tcenter*). Each individual neuron received the synchronous spike event at a time *Tcenter + r,* where *r* is a random number drawn from a uniform distribution (*U*[0,1]). Center stimulus was presented to the neurons in three conditions: center only, center stimulus just at the end of the surround stimulus (*see* below) and center while the surround stimulus was still on. *See* S3 Table for more details.

To mimic the surround stimulus, we selected 3,286 E-neurons and 3,286 I-neurons from a square region in the network with a side of 60 grid points. The center neurons (*see* above) were located at the center of this square. For the surround stimulus, we excluded the center neurons. The surround stimulus was injected in the selected neurons in the form of uncorrelated Poisson-type spike trains. *See* S3 Table for more details. The network model was simulated using the simulator NEST [45].

To quantify these network interactions, we used a spiking neuron model with conductance-based synapses, which were connected to each other based on physical distance according to a Gaussian connectivity kernel (*see* S3 Table). Because we were interested in the contribution of different connections, we kept the connection probability and distance-dependent connectivity kernel fixed and systematically varied the connection strengths (*see* S3 Table for stimulus input; S4 Table for neuronal parameters; S5 Table for synaptic parameters; S6 Table for network connectivity; and S7 Table for external input parameters). Consistent with experimental findings [46], the network remained in an inhibition-dominated activity regime in which inhibitory neurons displayed a higher background activity than excitatory neurons for all synaptic strengths (*see* S7A Fig).

To study the impact of *E–E* recurrent and their strength, we did two different simulation experiments. In the baseline model, *E–E* connection probability was set to 0.135. First, we reduced the *E–E* connection probability to 80%, 60%, and 40% of the baseline value. Next, for the case of 40% *E–E* connection probability, we increased the strength of *E–E* connection. All other model parameters remained unaffected. For these two simulation experiments, we followed the same procedure as for the results shown in Fig 4.

### Statistical analysis and data plotting

For each mouse, 10–16 sections were stained and visualized. Confocal images (20×) were used for the manual scoring of co-expression of vGAT/vGluT2, V5, and RV-GFP. Whole-brain images (10×) were acquired with a fluorescent microscope (Leica DM6000B) and a digital camera (Hamamatsu Orca-FLASH 4.0 C11440). They were manually registered to a bregma AP coordinate (from bregma −3.2 to −4.9), and subsequently aligned to the Allen Reference Atlas with the WholeBrain R package [47,48]. Every detected Rb-EGFP-positive neuron was registered, and the coordinates were used for the following analysis. As the main source of monosynaptic input and local input, only the RV-GFP-positive cells in the SCs were included in the calculation. For the comparison between mice, the number of input neurons were normalized to the total number of detected Rb-EGFP positive neurons in SCs. All the density estimations were done using a Gaussian kernel and were then plotted in R.

To minimize the effects of M-L differences of the injection sites between animals, the centroid of the starter neurons was first computed using k-means clustering analysis, which then allowed us to plot the relative M–L coordinate for each neuron. As for the depth estimation, the coordinates of the SC surface were first obtained with the WholeBrain R package, then the depth of each neuron was calculated after subtracting the difference. In both, M–L and depth density plots, only three central brain sections with the highest starter density were included according to the AP density plot.

After confocal imaging, the *cFos* fluorescence was quantified by a 3d object counter plug-in of Fiji. Colocalized *cFos*+ and GABA+ cells were manually counted in the brain section under the optic fiber implant (Figs 2G–2I and S6). The statistical analysis and visualization were done in Python.

We used three model stimulation protocols: center only (center), center stimulus just at the end of the surround stimulus (center after surround) and center while the surround stimulus was still on (center with surround). Each paradigm was repeated 20 times. For each stimulus presentation, we recorded excitatory conductance from a single neuron. We estimated the across-trial mean and variance of the conductance input to the recorded neuron. Finally, the results were rendered as the mean and variance of the excitatory conductance at specific time points after the onset of the stimulus in all three stimulus paradigms. In addition, we recorded spiking activity from all the neurons and estimated the firing rate of neurons. The network model was simulated using the simulator NEST. All differential equations were integrated using Runga–Kutta method with a time step of 0.1 ms.

Electrophysiology data analyses were performed using Clampfit 11 (Molecular Devices, USA), GraphPad Prism (GraphPad Software 7.00, USA), ImageJ (1.53c, NIH, USA), RStudio (R 3.6.1), or MATLAB (R2018b, MathWorks, USA). Data were all presented as mean values ± s.e.m. Before performing paired *t* test or one-way ANOVA analysis, the normality and the homogeneity of variance were first evaluated using Kolmogorov–Smirnov’s test and Brown–Forsythe’s test. If the *p* value of Kolmogorov–Smirnov’s test was less than 0.05, Wilcoxon’s signed-rank test would be used instead of paired *t* test. If *p* value of Brown–Forsythe’s test was less than 0.05, Friedman test and Kruskal–Wallis test would be used instead of a repeated-measures one-way ANOVA (RM one-way ANOVA) and ordinary one-way ANOVA, respectively. *p* values were corrected for deviation using the Geisser–Greenhouse correction. Once the *p* value of a one-way ANOVA analysis was less than 0.05, Tukey’s multiple comparisons test would be performed. *F* and df (df1, df2) are degrees of freedom for one-way ANOVA and paired *t* test, respectively. *p* values less than 0.05 were considered significant with asterisks in figures denoting as follows: *p < 0.05, **p < 0.01, ***P < 0.001, ****p < 0.0001, *ns*, no significance. Traces showing electrophysiological recordings plotted in the figures were NC for the liquid junction potential, which was experimentally found to be −9.8 mV (~−10 mV). It was taken, however, into consideration when estimating for their underlying evoked synaptic conductance.

**Keypoints**

- **Subcortical Visual Saliency**: The superficial superior colliculus (SCs) can actively contribute to visual saliency through local center-surround interactions, independent of cortical input.
- **Collicular surround suppression**: Patterned optogenetics combined with whole-cell recordings revealed how surround network activation can actively modulate the center excitability of single neurons, demonstrating the role of local SC circuits in surround modulation.
- **Recurrent Collicular Circuits:** *Trans*-synaptic mapping and modeling show that surround suppression depends on recurrent excitation and feedback inhibition, mirroring cortical connectivity architectures with deeper evolutionary origins.

## Supporting information

S1 Fig Mapping excitatory and inhibitory zones of SCs neurons (supplemental to Fig 2). **(A)** *(Top)* Procedure showing expression of ChR2 in RGCs using an intravitreal viral injection of the vector rAAV2-hsyn-hChR2(H134R)-EYFP (in WT mice, *N* = 14) and unilateral labeling of contralateral SCs inhibitory neurons with the vector rAAV5-FLEX-tdTomato (in this case vGAT-Cre mice were used, *N* = 5). *(Bottom)* Coronal midbrain section with ChR2-expressing RGC axonal terminals seen occupying the SCs. **(B)** Dual magnification setup for monosynaptic excitation zone mapping of single cells. This enabled the simultaneous visualization of a large field of view (FOV) encompassing the SCs (magenta rectangle in *A*), and single cell resolution (SCR) for identifying center excitation zones using whole-cell recordings and patterned optostimulation via a digital mirror device (DMD). *See* Materials and methods for more information. **(C, D)** A wide-field (WF) SCs neuron responding with excitatory and inhibitory postsynaptic currents (EPSCs measured at −65 mV shown in (C); IPSCs measured at 0 mV shown in (D) in response to a 20 Hz 5 ms-light pulse train delivered pseudo-randomly as a 100 × 70 μm grid optostimulation. Bottom panels: Response maps superimposed with the reconstructed morphology of the intracellularly stained WF neuron with Neurobiotin during whole-cell recording. Heat maps with darker shades coding for stronger responses in amplitudes. **(E)** Distance from soma as a function of normalized postsynaptic amplitude in this WF SCs neuron. Red for excitatory; Blue for inhibitory. Vertical dashed line indicates threshold applied at a normalized value of 0.2. **(F)** Center and surround zone of the WF zone determined by following thresholding approach applied to the excitatory PSCs shown in (C). Areas occupying a larger value compared to threshold are considered to be part of the center zone. *Abbreviations:* c., contralateral; comp, computer; Rec, recording; WT, wild-type; ChR2, channelrhodopsin-2; RGC, retinal ganglion cell; VIS, primary visual cortex; RSP, retrosplenial cortex; SCs, superior colliculus superficial layer; SCi, superior colliculus intermediate layer; SCd, superior colliculus deep layer; PAG, periaqueductal gray matter; LGN, lateral geniculate nucleus.
(EPS)

S2 Fig Three different examples of synaptic modulation between center and surround zones (supplemental to Fig 2). **(A)** *(Top)* Individual current traces held at reverse potentials for chloride-mediated GABAergic inhibition (−65 mV) and glutamate-mediated ionotropic excitation (0 mV) that span continuously surround and center optostimulation of the neuron (2 pulses separated by 20 ms) shown in Fig 2C and 2D. Here, surround network activation can be seen to effectively reduce the center EPSC amplitudes (cyan traces in blue rectangle) when comparing to center only responses (in magenta). Evoked responses to surround stimulation of all durations (5, 20, and 50 ms; shades of cyan) are also seen to drive bistable excitatory and inhibitory responses. *(Bottom)* Voltage-clamp (VC) recordings in response to surround optostimulation after bath application of 10 μM Gabazine (GBZ), thereby removing the effect of GABAergic inhibition. Same color-coding scheme as in Fig 2. Time stamps are centered around the onset of the first center pulse. **(B)** *(Top)* Synaptic current recordings performed in voltage-clamp of a stellate neuron that is subject to the 20 Hz optostimulation (2 pulses). Center EPSCs shown in the rectangle are truncated (dotted line). *(Bottom)* Same neuron recorded in current-clamp (CC) reveals the shunting effect that surround activation has on center responses (gray rectangle) when recording at −65 mV and at a depolarized membrane potential of −20 mV. This is attributed to chloride current triggered by the surround (revealed when held at 0 mV in VC; top traces). **(C)** Current traces from a vGAT+ SCs neuron in response to 20 Hz optostimulation (5 center pulses) that shows signs of surround suppression without the direct effect of synaptic inhibition. Top and middle traces: Current recordings performed at −65 mV and 0 mV reveals that this neuron does not receive direct inhibition from the surround yet its center response is attenuated suggesting that it receives less excitatory input. Bottom: Enlarged view of current traces found in blue rectangle in middle panel focusing on first and second paired pulse center stimulations. Surround stimulation has a significant effect on the first center pulse by reducing its amplitude by 50% (*** *p* < 0.001; *t* test). Note that liquid junction potential correction was not performed, which was experimentally measured to be ~ 10 mV.
(EPS)

S3 Fig Center-surround dynamics are defined by transient excitatory and inhibitory conductances (supplemental to Fig 2). **(A)** Total synaptic conductance (*G*_*T*_, black line) triggered by center only (C_1_) and center-surround (C_s_) conditions is decomposed into excitatory (*G*_*e*_, red) and inhibitory (*G*_*i*_, blue) conductances. Conductances belong to recording shown in Fig 2B–2E, which are estimated by Wehr and Zador (2003) by using current recordings performed at −65 mV and 0 mV that were corrected for the liquid junction potential. Time stamps *t*_*c*_, *t*_*e*_, and *t*_*T*_ symbolize the time onset for center stimulation, the time instant when peak excitation and maximal total conductance is reached, respectively. Solid lines are with center only condition and dashed lines are when center follows surround optostimulation. Red arrow indicates non-zero inhibitory conductance evoked by surround network activation at the time of *t*_*c*_. **(B)** Comparison of total synaptic conductance *G*_*T*_ between C_1_ and C_s_ conditions. Corresponding excitatory and inhibitory conductances at peak *G*_*T*_ are shown in Fig 2F and 2G. Color coding corresponds to morphologically or genetically identified SCs neuron cell-types (*see* Fig 2F). **(C)** Full time course of excitatory and inhibitory conductance during both stimulation conditions, C_1_ and C_s_. *Legends:* C-C (paired pulse center optostimulation) and S-C refers to surround followed by center optostimulation after a 20 ms refractory period. **(D)** Latency to peak inhibition as a function of latency to peak excitation. A relative delay *Δt* of 20 ms is observed when comparing the center (magenta) and surround (cyan) conditions. This suggests that the signals originating from the surround region interact with the center responses with this time difference. Curves are a gaussian probability distribution function fitted to the data points. The data underlying this figure can be found in the Supplementary section.
(EPS)

S4 Fig Identification of SCs cell types used for electrophysiology analysis (supplemental to Fig 2). **(A)** Reconstructed morphologies from various subtypes of SCs neurons that were recorded and presented in Fig 2, including wide-field, narrow-field, stellate and horizontal neurons, that were stained with Neurobiotin. **(B, C)** Quantification and classification of cell types according to morphology (i.e., horizontal, stellate, etc.) and genotype (i.e. vGAT+/− or random type). A total of *n = *41 were performed of which a subset of *n = *24 that maintained stability (stable access resistance over the period of the whole-cell recording that usually lasted 60 min or 120 min in the case that drugs were bath applied) were included in the analysis shown in Fig 2. **(D)** Relationship between dendritic field and the neuron’s corresponding retinorecipient center zone. Individual center zones typically exceed the area of the dendritic zone. Note that this correlation was performed for recorded neurons where morphological data were successfully obtained. The data underlying this figure can be found in the Supplementary section.
(EPS)

S5 Fig Classification of vGluT2 and vGAT cell types and their distribution across the SCs (supplemental to Fig 3). **(A)** Co-expression of *vGAT* and *vGluT2* in situ hybridization in the SCs using RNAscope in situ hybridization performed to reveal the identity of input neurons. White arrows indicate Rb-EGFP+ cells that are positive for *vGluT2* and negative for *vGAT*, suggesting that these input neurons are excitatory. By contrast blue arrows show Rb-EGFP+ cells that are inhibitory since they are positive for *vGAT* and negative for *vGluT2*. Scale bar: 25 μm. **(B)** The proportion of vGluT2+ (red, *n =* 70) and vGAT+ (blue, *n = *199) input cells that colocalize with Rb-EGFP. Unidentified neurons shown in purple (*n = *3/272). *N *=* *1 animal for vGAT and *N =* 1 for vGluT2, 3 sections were used per animal. Total number of counted neurons is: 272. **(C)** RNAscope in situ hybridization for identity confirmation of starter neurons (*see* white arrows). Top row: Colocalization of vGlut2 detected in cells that express Rb-EGFP and the Cre-inducible V5 tag expressed in vGluT2-Cre mice, confirm starter neurons are excitatory. Bottom row: The same approach applied in a vGAT-Cre. Colocalization Rb-EGFP+ with V5+ but negative for vGluT2, suggests that the starter neuron is putatively inhibitory. Scale bar: 25 μm. **(D)** The distribution of starter neurons in each animal, shown as proportion in different sublayers. **(E)** Schematic depicting the dorsoventral and mediolateral location of starter and input neurons in five separate anterior-posterior (AP) coronal sections of the SCs layer in a vGluT2-Cre animal. **(F)** Normalized density plot of starter and input neurons along anterior-posterior axis (AP; Left), medio-lateral axis (ML; Center) or depth from SC surface (Right). VGluT2+ and VGAT+ groups are shown in the top and bottom rows, respectively. The ML density plot is computed on the relative coordinates to the k-means centroid of starter cells. Dashed lines represent individual animals, and solid lines show the average. **(G)** *(Top)* Helper expression probability showing the percentage of neurons expressing V5 out all the vGAT (left, vGAT-Cre mice) and vGluT2 (right, vGluT2-Cre mice) neurons located within starter zone. *(Bottom)* Input expression probability for both vGAT and vGluT2 neurons inside and outside the starter zone. The probability is calculated on an *N = 3* for each genotype. The data underlying this figure can be found in the Supplementary section.
(EPS)

S6 Fig Functional recruitment of excitatory and inhibitory SCs neurons by RGCs (supplemental to Fig 3). **(A)** Pipeline that we used to measure in vivo *cFos* expression induced by local optostimulation of RGC axonal terminals in the SCs. Top left: As in Fig 2A, expression of ChR2 in RGCs was achieved using an intravitreal viral injection of the vector rAAV2-hsyn-hChR2(H134R)-EYFP (in WT mice, *N* = 5) or rAAV2-hsyn-mCherry for control (in WT mice, *N* = 3). Bottom left: schematic illustrating assay performed in a dark box. Right panels: cartoon of the optic fiber placement site shown in coronal (top) and horizontal (bottom) midbrain sections. **(B)** In vivo *cFos* expression resulting from optostimulation of RGC axonal terminals under the tip of the optic fiber implanted in the SCs (*see* Fig 3G and 3H). From top left: (a) merged image, (b) *cFos* staining, (c) GABA immunostaining (arrows show colocalization), and (d) ChR2-expressing retinal ganglion axonal terminals. ChR2 shown in magenta; *cFos* in yellow; GABA in cyan. Scale bar: 50 μm.
(EPS)

S7 Fig Complete raster of spiking network simulation (supplemental to Fig 4). **(A)** Network model simulation using E/I connectivity patterns summarized in Fig 4A. Raster of spiking activity from 6,400 excitatory and 6,400 inhibitory neurons with four stimulus conditions that are marked with vertical numbered lines that simulate experimental conditions shown in Fig 2. Conditions 1: center only before surround, 2: surround only, 3: center during surround, and 4: center after surround. **(B)** Expanded network response of conditions 1 (top) and 4 (bottom) shown in (A). **(C)** Spatial arrangement of center and surround network response profiles during the four different stimulus impulse conditions. The square schematically shows the region in which neurons were placed on a square grid. Excitatory (top) and inhibitory neurons (bottom) within the white circle received inputs corresponding to the center stimulation. All the neurons in white square but outside the inner circle received inputs corresponding to the surround network. Each subplot shows activity measured within a bin of 5 ms. **(D)** Spiking frequency of model inhibitory neurons as a function of the spiking frequency of excitatory neurons. The inverse relationship is correlated of the peak amplitude ratio of the “center during surround” divided by the “center-only” response (*see* color map).
(EPS)

S1 Data Raw numerical values underlying the figures and analyses presented in the manuscript. Each sheet corresponds to the dataset used for a specific figure inset.
(XLSX)

S1 Table Viruses used in this study. (DOCX)

S2 Table Drugs used in electrophysiology experiments. (DOCX)

S3 Table Stimulus input. (DOCX)

S4 Table Neuron parameters. (DOCX)

S5 Table Synapse parameters. (DOCX)

S6 Table Network connectivity. (DOCX)

S7 Table External Input. (DOCX)

## Abbreviations

AAV: adeno-associated virus
AP: anteroposterior
DMD: digital mirror device
DV: dorsoventral
*E–E*: excitatory-to-excitatory
EPSCs: excitatory postsynaptic currents
FOV: field of view
*I–E*: inhibitory-to-excitatory
IPSCs: inhibitory postsynaptic currents
ML: mediolateral
NC: not corrected
PBS: phosphate-buffered saline
PFA: paraformaldehyde
RG: rabies glycoprotein
RGC: retinal ganglion cell
SCs: superior colliculus
