# Supplementary material for: Recurrent circuits encode de novo visual center-surround computations in the mouse superior colliculus
Source: PLoS Biol. 2025 Oct 16;23(10):e3003414. doi: 10.1371/journal.pbio.3003414 (PMC12530612; doi:10.1371/journal.pbio.3003414)
Supplement: S1 Table — (DOCX) [file pbio.3003414.s009.docx]

**Supplementary Table 1. Viruses used in this study**

| Name | Type | Titer | Source |
| --- | --- | --- | --- |
| rAAV2/hsyn-hChR2(H134R)-EYFP-WPRE-PA | AAV2 | ﻿5.6 × 10¹² vg/mL | UNC Vector Core |
| rAAV2/hsyn-hChR2(H134R)-mCherry-WPRE-PA | AAV2 | ﻿5.1 × 10¹² vg/mL | UNC Vector Core |
| ﻿rAAV2-EF1α-DIO-hChR2(H134R)-EYFP | AAV2 | ﻿4.4 × 10¹² vg/mL | UNC Vector Core |
| ssAAV-2/2-shortCAG-dlox-hChR2(H134R)_EYFP(rev)-dlox-WPRE-hGHp(A) | AAV2 | ﻿1.4 × 10¹² vg/mL | ZNZ Viral Vector Facility |
| rAAV2/hsyn-mCherry | AAV2 | ﻿5.3 × 10¹² vg/mL | UNC Vector Core |
| rAAV5-CAG-FLEX-tdTomato | AAV5 | ﻿7.8 × 10¹² vg/mL | UNC Vector Core |
| AAV5-EF1a-DIO-TVA-V5-t2A-RG | AAV5 | 1.2 × 10¹² vg/mL | DMC laboratory |
| EnvA-ΔG-Rb-EGFP | Modified Rabies | 1 × 10¹º vg/mL | DMC laboratory |
