# Supplementary material for: Recurrent circuits encode de novo visual center-surround computations in the mouse superior colliculus
Source: PLoS Biol. 2025 Oct 16;23(10):e3003414. doi: 10.1371/journal.pbio.3003414 (PMC12530612; doi:10.1371/journal.pbio.3003414)
Supplement: S2 Table — (DOCX) [file pbio.3003414.s010.docx]

**Supplementary Table 2. Drugs used in electrophysiology experiments**

| Compound | Drug name | Concentration | Source |
| --- | --- | --- | --- |
| Gabazine  (SR 95531) | 6-Imino-3-(4-methoxyphenyl)-1(6*H*)-pyridazinebutanoic acid hydrobromide | 10 μM | Tocris Bioscience, USA |
| TTX | (4*R*,4a*R*,5*R*,7*S*,9*S*,10*S*,10a*R*,11*S*,12*S*)-Octahydro-12-(hydroxymethyl)-2-imino-5,9:7,10a-dimethano-10a*H*-[1,3]dioxocino[6,5-*d*]pyrimidine-4,7,10,11,12-pentol | 1 μM | Tocris Bioscience, USA |
| 4-AP | 4-Aminopyridine | 100 μM | Sigma-Aldrich |
| QX314 | *N*-(2,6-Dimethylphenylcarbamoylmethyl)triethylammonium bromide | 3 mM | Tocris Bioscience, USA |
