# Supplementary material for: Recurrent circuits encode de novo visual center-surround computations in the mouse superior colliculus
Source: PLoS Biol. 2025 Oct 16;23(10):e3003414. doi: 10.1371/journal.pbio.3003414 (PMC12530612; doi:10.1371/journal.pbio.3003414)
Supplement: S3 Table — (DOCX) [file pbio.3003414.s011.docx]

**Supplementary Table 3. Stimulus input**

| Type of input | Stimulated neurons | Spike input | Stimulus PSP | Repeats |
| --- | --- | --- | --- | --- |
| Center Input | All neurons (exc. and inh.) in a circle with radius 10 grid points.  314 exc. neurons and 314 inh. neurons. | Each neuron received 10 synchronous spikes corresponding to the presentation of a flash like center stimulus. Arrival time of each synchronous spike event was taken from a uniform distribution U[0,1] ms. | 1.03 mV at -54 mV to exc. neurons  0.86 mV at -54 mV to exc. neurons | 20 |
| Surround input | All neurons (exc. and inh.) within a square of size 60 grid point centered at the center of the network. Neurons part of the center stimulus were excluded.  3286 exc. neurons and 3286 inh. neurons. | Poisson type spiking input with a spike rate of 100 Hz.  Surround stimulus was presented for 200 ms. | 2.58 mV at -54 mV | 20 |
