# Supplementary material for: Recurrent circuits encode de novo visual center-surround computations in the mouse superior colliculus
Source: PLoS Biol. 2025 Oct 16;23(10):e3003414. doi: 10.1371/journal.pbio.3003414 (PMC12530612; doi:10.1371/journal.pbio.3003414)
Supplement: S4 Table — (DOCX) [file pbio.3003414.s012.docx]

**Supplementary Table 4. Neuron parameters**

| Parameter | Value | Description |
| --- | --- | --- |
| *C_m_* | 200 pF | Membrane capacitance |
| *g_L_* | 20 nS | Leak conductance |
| *V_th_* | -54 mV | Spike threshold |
| *E_L_* | -70 mV | Reversal potential for leak conductance |
| *t_ref_* | 2 ms | Refractory time |
