# Supplementary material for: Recurrent circuits encode de novo visual center-surround computations in the mouse superior colliculus
Source: PLoS Biol. 2025 Oct 16;23(10):e3003414. doi: 10.1371/journal.pbio.3003414 (PMC12530612; doi:10.1371/journal.pbio.3003414)
Supplement: S5 Table — (DOCX) [file pbio.3003414.s013.docx]

**Supplementary Table 5. Synapse parameters**

| Parameter | Value | Description | Notes |
| --- | --- | --- | --- |
| *J_ex_* | 0.63 mV | EPSP amplitude for connection from external excitatory inputs | EPSP amplitude was measured at a holding potential of -54 mV |
| *J_ix_* | -0.3 mV | IPSP amplitude for connection from external inhibitory inputs | IPSP amplitude was measured at a holding potential of -54 mV |
| *J_ee_* | {0.13, 0.26} mV | EPSP amplitude for exc. → exc. connection | EPSP amplitude was measured at a holding potential of -54 mV |
| *J_ei_* | {0.11, 0.21, 0.32, 0.42, 0.53, 0.63} mV | EPSP amplitude for exc. → inh. connection | EPSP amplitude was measured at a holding potential of -54 mV |
| *J_ie_* | {-0.14,-0.17, -0.19, -0.22, -0.28, -0.3} mV | IPSP amplitude for inh. → exc. connection | IPSP amplitude was measured at a holding potential of -54 mV |
| *J_ii_* | -0.055 mV | IPSP amplitude for inh. → inh. connection | IPSP amplitude was measured at a holding potential of -54 mV |
| *tau_e_* | 1.0 ms | Time constant of excitatory synapses | This was the same for both exc. → exc., exc. → inh. synapses and external excitatory input synapses |
| *tau_i_* | 3.0 ms | Time constant of inhibitory synapses | This was the same for both inh. → exc., inh. → inh. synapses and external inhibitory input synapses |
| *d_ee_* | 0.2 ms | Synaptic delay for exc. → exc. synapses |  |
| *d_ei_* | 0.1 ms | Synaptic delay for exc. → inh. synapses |  |
| *d_ie_* | 0.1 ms | Synaptic delay for inh. → exc. synapses |  |
| *d_ii_* | 0.1 ms | Synaptic delay for inh. → inh. synapses |  |
