# Supplementary material for: Recurrent circuits encode de novo visual center-surround computations in the mouse superior colliculus
Source: PLoS Biol. 2025 Oct 16;23(10):e3003414. doi: 10.1371/journal.pbio.3003414 (PMC12530612; doi:10.1371/journal.pbio.3003414)
Supplement: S6 Table — (DOCX) [file pbio.3003414.s014.docx]

**Supplementary Table 6. Network connectivity**

| Parameter | Value | Description | Notes |
| --- | --- | --- | --- |
| *N_e_* | 6400 | Excitatory neurons |  |
| *N_i_* | 6400 | Inhibitory neurons |  |
| Excitatory grid size | 80 × 80 | Neurons were placed on a regular grid |  |
| Inhibitory grid size | 80 × 80 | Neurons were placed on a regular grid |  |
| *C_ee_* | 0.135 | Connection probability between exc. to exc. neurons. This number refers to the integral of the connectivity kernel | Based on our experimental data (*See* Fig. 3K) |
| *C_ei_* | 0.112 | Connection probability between exc. to inh neurons. This number refers to the integral of the connectivity kernel | Based on our experimental data (*See* Fig. 3K) |
| *C_ie_* | 0.315 | Connection probability between inh to exc. neurons. This number refers to the integral of the connectivity kernel | Based on our experimental data (*See* Fig. 3K) |
| *C_ii_* | 0.3375 | Connection probability between inh. to inh. neurons. This number refers to the integral of the connectivity kernel | Based on our experimental data (*See* Fig. 3K) |
| *S_e_* | 16 grid points  2% of the network size | Standard deviation of Gaussian used to estimate distance dependent connectivity from exc. to exc. and exc. to inh. neurons. |  |
| *S_i_* | 20 grid points  2.5% of the network size | Standard deviation of Gaussian used to estimate distance dependent connectivity from inh. to exc. and inh. to inh. neurons. |  |
