# Supplementary material for: Recurrent circuits encode de novo visual center-surround computations in the mouse superior colliculus
Source: PLoS Biol. 2025 Oct 16;23(10):e3003414. doi: 10.1371/journal.pbio.3003414 (PMC12530612; doi:10.1371/journal.pbio.3003414)
Supplement: S7 Table — (DOCX) [file pbio.3003414.s015.docx]

**Supplementary Table 7. External Input**

| Parameter | Value | Description |
| --- | --- | --- |
| nu_exc | 1000 Hz | Poisson type spike trains were injected in all neurons |
| nu_inh | 600 Hz | Poisson type spike trains were injected in all neurons |
